# Understanding maternal choices and experiences of care by skilled providers: Voices of mothers who delivered at home in selected communities of Lusaka city, Zambia

**DOI:** 10.3389/fgwh.2022.916826

**Published:** 2023-01-06

**Authors:** Choolwe Jacobs, Charles Michelo, Adnan Hyder

**Affiliations:** ^1^ School of Public Health, University of Zambia, Lusaka, Zambia; ^2^ Harvest Research Institutes, Harvest University, Lusaka, Zambia; ^3^ Milken Institute School of Public Health, George Washington University, Washington, DC, United States

**Keywords:** skilled birth attendance, childbirth, urban slums, maternal choices, Zambia

## Abstract

**Background:**

Significant proportions of women living in urban areas including the capital cities continue to deliver at home. We aimed to understand why mothers in a selected densely populated community of Lusaka city in Zambia deliver from home without assistance from a skilled provider during childbirth.

**Methods:**

Using a phenomenological case study design, we conducted Focus Group Discussions and In-depth Interviews with mothers who delivered at home without assistance from a skilled provider. The study was conducted between November 2020 and January 2021 among 19 participants. Data were analysed using content analysis.

**Results:**

Individual-related factors including the belief that childbirth is a natural and easy process that did not require assistance, lack of transport to get to the health facility, influence and preference for care from older women who were perceived to have the experience and better care, failure to afford baby supplies, and waiting for partner to provide the supplies that were required at the health facility influenced mothers’ choices to seek care from skilled providers. Health system-related factors included mistreatment and disrespectful care such as verbal and physical abuse by skilled healthcare providers, stigma and discrimination, institutional fines, and guidelines such as need to attend antenatal care with a spouse and need to provide health facility demanded supplies.

**Conclusion:**

Individual and health system access related factors largely drive the choice to involve skilled providers during childbirth. The socioeconomic position particularly contributes to limited decision-making autonomy of mothers, thus, creating challenges to accessing care in health facilities. The health system-related factors found in this study such as mistreatment and disrespectful care suggests the need for redesigning effective and sustainable urban resource-limited context maternal health strategies that are culturally acceptable, non-discriminatory, and locally responsive and inclusive. Rethinking these strategies this way has the potential to strengthening equitable responsive health systems that could accelerate attainment of sustainable developmental goal (SDG) 3 targets.

## Introduction

Access to quality facility-based services by skilled birth attendants during pregnancy, delivery, and post-delivery is associated with improved maternal health outcomes (1, 2). However, despite efforts being made to improve coverage for Skilled Birth Attendance (SBA) by mothers during childbirth is lower than the ideal in most developing countries, including Zambia. Disparities in utilisation of the services also exist, with the rural populations being more disadvantaged. However, although rural populations are generally known to be more disadvantaged and less likely to access the services than the urban population (3), some poor populations in the urban areas including the capital cities are increasingly becoming disadvantaged with low coverage for SBA (4).

Skilled birth attendance is one of the important interventions along the continuum of care. According to the World Health Organization (WHO), a skilled birth attendant is “an accredited health professional such as a midwife, doctor or nurse-who has been educated and trained to proficiency in the skills needed to manage normal (uncomplicated) pregnancies, childbirth and the immediate postnatal period, and in the identification, management and referral of complications in women and newborns” (5, 6). Having a skilled birth attendant with midwifery skills present at childbirth has been shown to reduce maternal and neonatal morbidity and mortality (2). However, despite the significant role of SBAs for provision of quality care to the mother and her baby, previous studies have reported barriers to the use of SBAs during childbirth by mothers (3, 5). In Zambia, maternal health delivery including SBA is among the national health service priorities, and the services are provided at no cost for all women in public health.

Barriers to access to care by skilled providers during childbirth include health system-related factors such as lack of resources, disrespectful and abusive maternity care (6), and limited availability of healthcare workers (7–10), while individual-related factors include mother's age (11), marital status (12), level of education (13), cultural beliefs, and economic position (14–16). Although some studies have also reported willingness by women to seek care from SBAs (17), women's perceptions and experiences with care provided by SBAs during childbirth, such as disrespectful and abusive care (18, 19), influence their health-seeking behaviours, thus contributing to the existing disparities to access and utilisation of care from SBAs during childbirth.

Understanding the experiences and perceptions of those who do not access care from skilled providers during childbirth is important to inform policy and practice. In particular, the voices of women that deliver from home are important to fully understand the drivers that influence their critical choices. However, evidence on mothers’ choices and experiences in urban areas particularly in selected densely populated communities is limited. Therefore, we aimed to understand why mothers in a selected community of Lusaka city deliver from home without assistance from a skilled provider during childbirth. Specifically, we qualitatively (1) explored individual- and family-related factors that influence mothers to make their choices and perspectives about care during childbirth from skilled providers; and (2) explored health system-related factors and experiences of care during childbirth from a skilled provider.

## Andersen's behavioural model

This study conceptualised the individual- and family-related and health system-related factors that influence mothers to make their choices and perspectives about care during childbirth from skilled providers based on Andersen's framework (20). Andersen's behavioural model is a logical explanatory framework of how use of services can be regarded as a type of individual behaviour. This framework postulates that some population, individual, and health system characteristics contribute to an individual's use of health services. According to this framework, health system characteristics such as policies, environments, and resources (human and financial) and health behaviour characteristics that include individual health choices of a woman, based on personal perceptions and preferences over the services provided, influence accessibility of health services.

## Methods

### Design, setting, and population

We employed a phenomenological case study design between November 2020 and January 2021. Specifically, we qualitatively explored participants’ experiences and perceptions within their context (21–23) about the care from skilled providers during childbirth. Focus group discussions (FGDs) and in-depth interviews (IDIs) were conducted with mothers. Focus group discussions enabled rich data description because participants were able to openly share their experiences.

The study was conducted in the Kanyama suburb, an unplanned and densely populated peri-urban community of Lusaka, Zambia. Kanyama is located on the western peripheral of the city of Lusaka and has sprawled wider over the years. From the 2010 census, the population for Kanyama settlement was approximately 153,624, of which 49.9% of this population were females (24). This community was purposively selected because of its consistently low maternal health outcomes and poor SBA coverage. Most household heads in Kanyama community are not employed (24). They depend on small businesses selling on the streets and in the market for their income.

The primary study population was mothers with a child, having been born within 1 year period preceding the interview and delivered at home without assistance from a skilled provider. The mother needed to be aged between 18 and 49 years, living within the study community during their most recent pregnancy and delivery and birthed outside the health facility, without assistance by a skilled birth attendant.

### Recruitment procedures and sample size

Using the set heterogeneous selection strategy, eligible participants who expressed interest in the study were provided detailed information from the information sheet and those that consented were enrolled into the study (25). The recruitment of respondents was conducted within the communities with the help of community health workers (CHWs). Mothers who delivered from home were identified by CHWs and invited to participate in the study from their homes. This process continued until the minimum number of participants was attained and saturation was achieved. A total of 38 participants were interviewed.

### Data collection

Two data collectors (research assistants) with public health background and extensive experience in qualitative data collection conducted the interviews in November 2020. The research assistants underwent a 1-day training prior to data collection and were supervised by one of the co-authors (CJ). One research assistant facilitated the sessions, while the other one managed the audio recordings and took field notes. FGDs and IDIs were conducted in private areas, mostly preferred by the participants to ensure privacy and confidentiality (25). Triangulation of FGDs and IDIs was done to ensure trustworthiness and credibility of the data. Interview guides with core questions were developed by the authors, translated by a qualified translator and used to facilitate the interviews. The guides contained questions around experiences and perceptions mothers faced to seeking care from SBAs during childbirth. Focus group discussions were organised and conducted with six to eight participants in each. All interviews and discussions were conducted in common local languages (Nyanja or Bemba) and were digitally recorded. Additionally, field notes were taken by a note taker during the interviews. The data collection tools were piloted in a similar facility not included in the study to identify potential deficiencies in the research tools and ensure that respondents understand the questions in the same way (25). The average duration of FGDs and IDIs was 45 min.

### Transcription and analysis

Recorded interviews/discussions were transcribed verbatim in the local languages and then translated to English by research assistants. Some transcripts were randomly selected and verified by back translation into Nyanja for accuracy. The transcribed documents in Microsoft Word were thoroughly read and re-read to develop a coding scheme. Coding was completed by going line by line through the material and with the help of a research assistant, whereby each transcript was independently coded by two people for consistency. The codes agreed upon were organised to create categories and later themes (25). An inductive content analysis approach was employed to analyse the qualitative data. Triangulation of the different sources, including FGDs, IDIs, and field notes was employed to validate the data by using cross-referencing (26). The study credibility, where the results of the research are closely related to reality, was achieved through prolonged engagement, triangulation, peer debriefing, and member check. Prolonged engagement involves establishing adequate contact with the participants and the context with the objective of acquiring data the researchers need.

### Ethical considerations

To conduct this study, ethical clearance was secured from the University of Zambia Biomedical Research Ethics Committee (Ref. No. 913-2020). Permission was obtained from the Ministry of Health at different levels. Written informed consent was acquired from participants who could read and write, and fingerprints were used for participants unable to read and write. Anonymity and confidentiality were assured by ensuring that no names were used during interviews and on any information collected from participants. Interviews were conducted in safe, quiet, and comfortable places that were chosen by the participants, within the community. We conducted pretesting of the preliminary FGD guides with each of the research participants who were excluded from the actual data collection. This helped in estimating the time required to conduct the interviews and FGDs, to refine the interview guides and questions, to check appropriateness of the data capturing procedures, and to familiarise the researcher with the data recording equipment.

## Findings

### Participant distribution

Overall, we conducted 12 interviews/sessions (4 IDIs and 2 FGDs in each age category: 18–24 years and 25 years and above) with a total of 38 participants. Twenty-one (52%) of these mothers were married and living with their husbands at the time of the study. Only nine (27%) of the mothers had at least secondary education, while the rest of the participants had basic primary education. All the mothers had delivered their most recent child at home. Table 1 provides background information of the participants.

**Table 1:** Background information of participants.

Description of participants	18–24 years	25 years and above	Total number (%)	Number of respondents per interview
Type of interview				
Focus group discussion	2	2	4	6–8
In-depth interviews	4	4	8	1
Marital status				
Married	11	10	21 (52%)	N/A
Not married	8	9	17 (48%)	
Level of education				
Primary school	15	14	29 (63%)	N/A
Secondary school	4	5	9 (27%)	
Parity				
1–2 Children	12	4	16 (42%)	N/A
2–4 Children	7	9	16 (42%)	
More than 4 children	0	6	6 (16%)	

Data analysis revealed two major themes that explain factors influencing choices to care seeking for Skilled Birth Attendance during childbirth among mothers in a selected populated community of an urban city and these included (i) individual and family factors and (ii) health system factors (see Table 2).

**Table 2:** Factors to care seeking for skilled birth attendance during childbirth among mothers in a selected populated community of an urban city.

Themes	Sub-themes
Individual family-related factors	Belief that childbirth is an easy process
	Birth companionship and support at home
	Lack of transport to get to the health facility
	Influence from older women
	Perceived experience and better care by older women or TBAs
	Failure to afford or access baby supplies
Health system-related factors	Mistreatment and disrespectful care • Verbal abuse by skilled healthcare providers • Physical abuse • Discrimination and stigma
	Institutional rules and sanctions • Failure to attend ANC with spouse • Inability to afford hospital demanded supplies
	Poor environment of the labour ward

ANC, antenatal care.

## Individual- and family-related factors

### Belief that childbirth is an easy process

Some mothers decided to deliver from home because they perceived childbirth as an easy process that did not require assistance. Some older mothers narrated how they managed to deliver their most recent child at home successfully without assistance from a skilled provider and how help for assistance was called for from some elderly women within the community. One of the mothers who has always been delivering from home for all her children reported that she only calls for help to have the umbilical cord cut.

*I gave birth alone and only called my neighbour to help me cut the umbilical cord. For all my children, I have never had any problem because for me it is always not a problem. I manage to deliver on my own*. **FGD, Mother, 39 years**

Another mother explained how the labour process was a quick process for her such that she did not have time to get to the health facility.

*Let me talk about my pregnancies, mine is different, for me when labour comes, it does not give me chance to even prepare to get to the health facility because it comes so quickly and within a short time I deliver*. **FGD, Mother, 36 years**

### Lack of transport to get to the health facility

Challenges to get transport came out frequently from the mothers. Some mothers explained how delays to get transport led to them delivering from home. When asked why this was a problem, some mothers indicated that they needed to look for some money to pay for the transport to take them to the health facility for childbirth. One of the mothers indicated that while the husband went to look for transport, labour she had already delivered from home.

*I decided to give birth from home due to the fact that I didn't have transport to go to the clinic*. **FGD, Mother, 22 years**

### Influence from older women

Mothers who delivered from home without assistance from a skilled provider reported having been exposed to close relatives who were conducting deliveries from home, which led to them gaining skills and experience in delivering without skilled assistance. One mother explained how her grandmother she grew up with taught her how to go about the process of delivery and how to cut the umbilical cord and have the placenta expelled.

*I grew up with my grandmother, so I was educated on how to give birth and take care of the placenta and the child after delivering*. **IDI, Mother, 29 years**

Younger women in particular reported being recommended by their parents and guardians to deliver from home. This was common especially when the women had not adequately prepared for health facility delivery. One woman had this to say

*……my mum called for her friends to come and help me deliver*. **FGD, Mother, 19 years**

### Perceived experience and better care by older women or TBAs

The care provided by older women and some traditional birth attendants (TBAs) within the community during childbirth was perceived to be better than the care that mothers get in the health facilities by skilled providers. Some women went ahead with unassisted home birth as they believed that the care provided at home by older women was better than the care at the health facility by healthcare providers. Being delivered by neighbours or a TBA was considered a better because they and their babies were well taken care of during the process.

*I chose for myself because I know they help people and she was an experienced old lady*. **IDI, Mother, 21 years**

*For me she's my aunty, and I know she does that, she also helped my sister when she was pregnant. I called her; she helps in the family*. **FGD, Mother, 25 years**

### Birth companionship and support at home

Mothers also perceived birthing experience at home being better in comparison to the health facilities because of an opportunity to have their loved ones around them during birthing process. One mother narrated how her mother and elder sister were available throughout the process. Another mother reported being helped with wrapping and carrying her baby to the hospital immediately after giving birth. And this is what she had to say:

*For me it was comforting and in some way a relief to have my mother and my elder sister throughout the process, encouraging me that it was going to be over soon and also coddling me*. **FGD, Mother, 22 years**

### Failure to afford or access baby supplies

Generally, most women that delivered from home reported challenges in accessing the supplies such as disinfectants and baby layette required at the health facility due to inability to afford them. Because of this fact, most mothers were not prepared for skilled delivery particularly because they could not afford a black plastic used to spread on the delivery bed and some “jik” used as disinfectant. Some women believed that giving birth should not be costly as they could not afford.

*What made me give birth from home was because the baby's clothes weren't enough as well as the buckets*. **FGD, Mother, 26 years**

*I never had a plastic and JIK, so my mum called her friends to come and help me deliver*. **FGD, Mother, 25 years**

Some women waited for their partners to provide the supplies and baby layette that were required at the health facility. Dependence on the spouse/partner disadvantaged these women who could not provide.

*He was around but stepped out to look for a car and plastic in order to rush me to the clinic and when he had come back, he found I had already given birth*. **FGD, Mother, 22 years**

## Health system-related factors

### Mistreatment and disrespectful care

#### Verbal abuse by skilled healthcare providers

Mothers reported healthcare providers shouting, speaking harshly, and being rude to women during labour. Verbal abuse was reported by almost all the mothers including those that had delivered their first child from home. Mothers who had more than one child had reported their experiences giving birth from the health facility and how skilled birth attendants often mocked and shouted at them or at other patients in harsh tones. Mothers reported how verbal abuse was a particular experience when they failed to bring required supplies during delivery, while others it was reported that it was not spacing their children.

*The nurse on duty did not even care how I felt about the pain and my emotions as she kept on screaming using very bad words, all because I did not bring a plastic and some Jik to the labour ward*. **FGD, Mother, 26 years**

Some mothers felt that some female healthcare providers were rude compared to male healthcare providers. One of the mothers had this to say:

*Most male nurses show compassion, and the time I went to the clinic the female nurses that attended to me yelled at me thus discouraging me to give birth from the clinic*. **IDI, Mother, 22 years**

#### Physical abuse

Some mothers also explained how they were physically abused by some healthcare providers through slaps and pitches on the legs for not following the instructions. Several mothers also mentioned hearing reports of abuse or witnessing physical abuse of other mothers. One mother of four children explained how she was slapped by a healthcare provider during childbirth of her first child for not complying with instructions.

*I would like to add on, why we refrain from going to clinics is because the nurses don't show compassion, like the time I had my fifth child, the nurse slapped me.* **FGD, Mother, 42 years**

Mothers felt that nurses needed to explain the process to the mothers rather than being slapped.

#### Discrimination and stigma

Discrimination was another commonly reported form of mistreatment. Mothers felt judged because of their specific characteristics such as their level of education, HIV status, age, parity, and socioeconomic position. Women who had birthed more than four children were particularly mocked and judged for not taking contraceptives for birth control. One mother said

*Especially some of us with more children, they even scream more and scold you for not taking contraceptives*. **IDI, Mother, 22 years**

Another mother had this to say:

*For me I was in pain and was calling for help and one nurse was ignoring me and even said that I was exaggerating the pain because other women giving birth for the first time are calm. This constant reminder made me feel humiliated.* **FGD, Mother, 38 years**

Generally, mothers felt that such discrimination led them to receive substandard levels of care from skilled providers. Women also observed favouritism, particularly for women who seemed economically sound by presentation and those with an existing relationship with a healthcare provider. Some women went to an extent of offering small gifts to health workers to win their favour and better treatment.

### Institutional rules and sanctions

#### Failure to attend ANC with spouse

Some institutional rules and sanctions by the local health facility staff such as need to attend the first antenatal care (ANC) visit with a partner were a barrier for mothers to skilled birth attendance at the health institution. Some mothers who did not have partners to come with for ANC services avoided delivering from the health facility by a skilled healthcare provider because of fear of being chased or scolded by the healthcare providers.

*Aaaah… without having attended ANC? They will not leave you. Like I mentioned for me, my man refused that he was not the one responsible, so that made me not attend ANC and I could not even deliver from the health facility. They would have shouted at me*. **FGD, Mother, 26 years**

*When going for antenatal check-ups nurses ask us to go with our husbands, mostly our husbands do not want to attend the check-ups and nurses get upset and in the end we get shouted at when we go for delivery*. **FGD, Mother, 24 years**

One mother confirmed how she avoided delivering from a health facility by an SBA due to fear of being shamed and sent back because she never went with her husband during ANC.

*No one influenced me to give birth from home, I just had fear that if I went to the clinic I'd be sent back home because my husband didn't come with me during antenatal care*. **IDI, Mother, 25 years**

#### Hospital demanded supplies

Failure to afford the supplies, including disinfectants and baby clothes required by healthcare providers at the health facility, made mothers stay away from assisted childbirth by skilled healthcare providers. Narratives from mothers revealed that the requirements at the clinics were too much to afford. One of the mothers had this to say:

*I delivered from home because things were not enough for the baby, I never had JIK, a plastic and even the cloths for the baby were not there, so my mum called her friends to come and help me deliver*. **FGD, Mother, 22 years**

*What made me not deliver from the clinic was due to not having sufficient requirements needed by the clinic because what the clinic requires us to buy are way too many and when you're not able to meet their requirements you end up getting shouted at*. **FGD, Mother, 24 years**

Mothers did not understand why the need to provide the supplies was a priority on arrival to an extent that a mother had to provide the supplies before being attended to or she risked being sent away or screamed at.

### Poor environment of the labour ward

Mothers perceived the environment in the public facility being unconducive due to overcrowding with limited beds as well as poor sanitation. Some indicated how the toilets were dirty and could not be comfortably used.

*Aah, let me comment on the toilets, people complain about the toilets and the bathrooms, the clinics are always dirty. You don't even want to stay a second in there*. **FGD, Mother, 24 years**

## Discussion

Childbirth by a skilled provider has the potential to avert negative health complications to the mother and the child (5). The primary focus of our study was to understand reasons women in an urban community of Lusaka city of Zambia deliver from home without assistance from a skilled provider. Overall, we observed that mothers’ socioeconomic positions and health system access related factors largely drive the choice to involve skilled health care providers during childbirth. This includes individual and family factors such as cultural beliefs about childbirth, unaffordable childbirth-related costs (such as transport costs and costs for baby clothing and other supplies), dependence on the spouse, and older women to help make decisions about care seeking for childbirth that influenced mothers’ decision to deliver at home without support from a skilled provider. In addition to this and consistent to other studies (10, 11, 18), health system-related factors observed, including, but not limited to, institutional sanctions, mistreatment and disrespectful care, and poor attitudes of providers, created negative healthcare stereotypes that hindered women from uptake of care from skilled provider during childbirth.

The finding that individual and family factors such as cultural beliefs that childbirth is a natural and easy process that may not require assistance at the health facility is consistent to evidence that has reported that some women consider childbirth as a natural process that requires a personal responsibility (27, 28). Although pain during childbirth is one of the concerns for most women in this study and other similar studies (29), women believe in their own strength in managing pain and that they needed help to encourage and support enduring the pain and have the umbilical cord cut (28). The concerns on the health of the just born child demonstrated by almost all women rushing to the health facility to check if the child was fine implies that mothers care about the health of their newly born child and understand the need to seek care from skilled personnel at the health facilities (30). This finding suggests the need for demand-creation strategies for skilled health care providers that are rooted within complex sociocultural contexts and influence health-seeking behaviours. A better understanding of this urban resource-limited context is useful in refocusing effective and sustainable strategies that are culturally acceptable and locally responsive (31).

In this setting, we found that family members and older women impacted the decision-making process for seeking care from skilled birth attendants during childbirth. Our findings align with previous research that has reported mothers’ preference and confidence in elderly women to support childbirth at home (32, 33). The role of significant matriarchal figures locally known as “bana chimbusa,” continue to form an important community linkage for pregnancy and delivery services as they have continued facilitating home delivery among women in this urban context. Strategies in urban areas, especially in urban slums, should target social networks such as elderly women and other social networks that play critical roles during the pregnancy and birthing process in the communities that need to be enhanced through participatory approaches that engage social networks and through behavioural change messages for women. Furthermore, the socioeconomic dependence of women on their spouses observed in this study is in agreement with other studies (34–36) and implies that women in this setting are relatively poor members who cannot afford to pay for transportation on the onset of labour. These findings suggest an urgent need to address the gender inequities and empower women to be able to respond to their health needs and related costs without having to wait for their spouses (37).

Health system-related factors such as the attitudes of skilled healthcare workers found in this study attributed to the poor use of health facility-related childbirth. Similar to other studies (10, 38), in this study, women preferred to deliver from home because of discriminatory and disrespectful healthcare by the skilled providers. They believed that the relationship between themselves and SBAs needed be mutual and trustful. Despite the existing WHO intrapartum care guidelines (39), recommending respectful maternity care for all women, mothers in this study had no confidence in the health system and were fearful to deliver in the facility without their close relation. This finding is similar to other studies (5, 18, 40, 41). Physical abuse such as hitting and rough handling of the body parts and verbal abuse such as yelling or screaming, name calling, and threatening of women by skilled health care providers, narrated in this study, has been reported in another study too (11). Finding that women felt discriminated during childbirth in the labour ward mainly due to their pre-existing conditions such as HIV, socioeconomic status, parity, and age suggests the need for inclusive models. Furthermore, the complex drivers of mistreatment and disrespectful care during facility-based childbirth reported in this study suggests the need for interventions at the interpersonal level between mothers and their healthcare providers, to improve women's experience of care during childbirth. Effective communication and engagement strategies among healthcare providers, mothers, and their social networks are essential to ensure that care is responsive to mother's needs and preferences in all contexts (42).

This study also found that health facility rules and sanctions created by healthcare providers at local health facilities form an important part of mothers’ lived experiences on childbirth-related barriers. This finding is consistent with evidence in rural settings (43–45). Finding that mothers with fewer social and financial resources were not able to deliver from a health facilities by a SBA due to experiences and fear of being subjected to sanctions implies the inequitable effects that local institutional sanctions may have particularly among mothers who are unable to meet the requirements or have to make significant sacrifices to follow the rules (46). For instance, we found that mothers who did not attend ANC or did not come with a spouse during ANC including mothers who did not bring some disinfectant and a “black plastic” to the labour ward risked being shamed and mistreated during childbirth and so avoided care from skilled health care providers.

The reported institutional sanctions by the health system not only exclude vulnerable and poor women with insufficient financial and social resources but also reinforce inequitable provision of quality maternal healthcare services. This finding, therefore, suggests the need to adequately engage healthcare providers in enhancing health equity as they have a central role in addressing action on the social determinants of health and in promoting health equity. Research is also required to establish existence and implications of institutional rules and sanctions including understanding health workforce's current state and its role in health disparities.

A few limitations worth noting in this study include self-reporting of disrespectful care and mistreatment, which makes it difficult to identify underreporting and overreporting considering the sensitivity of the subject. In this study, we did not have the opportunity to validate the findings with participants due to COVID-19 restrictions. Nevertheless, the findings in this study remain important due to the data collection approach used by interviewing mothers within the community, which enabled open discussions within mothers’ own environments. The narratives from mothers with live experiences and delivering from home without an SBA make the findings more informative.

## Conclusion

We conclude that mothers’ individual positions and health system access related factors largely drive the choice to involve SBAs during childbirth. The socioeconomic position particularly might be the enabling environment giving room for the limited decision-making autonomy of mothers, thus, creating challenges to accessing care in health facilities. Importantly, some health system-related factors found in this study such as mistreatment and disrespectful care suggests need for redesigning effective and sustainable urban resource-limited context maternal health strategies that are culturally acceptable, non-discriminatory and locally responsive and inclusive. Overall, these observations suggest a need to redesign strategies that equitably expand coverage for SBA particularly in resource-limited urban areas so as to reach most at-risk populations, as an ethical responsibility. Rethinking these strategies this way has the potential to equitably expand service coverage sustainably and this could accelerate the attainment of sustainable developmental goal (SDG) 3 targets. Development of policies and guidelines that actively seek to interrupt such social marginalisation could contribute to strengthening equitable responsive health systems

## Data Availability

The raw data supporting the conclusions of this article will be made available by the authors, without undue reservation.
